# Peer Review of “Identifying Safeguards Disabled by Epstein-Barr Virus Infections in Genomes From Patients With Breast Cancer: Chromosomal Bioinformatics Analysis”

**DOI:** 10.2196/70041

**Published:** 2025-01-29

**Authors:** Anonymous

**Keywords:** breast, cancer, oncology, ovarian, virus, viral, Epstein-Barr, herpes, bioinformatics, chromosome, gene, genetic, chromosomal, DNA, genomic, BRCA, metastasis, biology

*This is the peer-review report for “Identifying Safeguards Disabled by Epstein-Barr Virus Infections in Genomes From Patients With Breast Cancer: Chromosomal Bioinformatics Analysis.”*

## Round 1 Review

Dear Author,

After a thorough review of the paper titled “Herpesvirus infections eliminate safeguards against breast cancer and its metastasis: comparable to hereditary breast cancers” [1] by Bernard Friedenson, here is the negative feedback and evaluation, along with a recommendation for the inclusion of a specific article in the discussion section.

### Negative Feedback and Evaluation

#### Clarity and Scope

The paper ambitiously attempts to link Epstein-Barr virus (EBV) infections to breast cancer development and metastasis. While the hypothesis is intriguing, the narrative sometimes lacks clarity and could benefit from a more focused scope. The vast amount of data and the complex mechanisms presented can be overwhelming and occasionally detract from the main message.

#### Methodological Concerns

The reliance on bioinformatics analyses and previously published datasets raises questions about the direct experimental validation of the proposed mechanisms. Although the computational approach is valid, the absence of direct experimental evidence or validation in breast cancer samples limits the strength of the conclusions.

#### Interpretation of Data

The interpretation of viral homology and its impact on cancer development is speculative in several sections. The connections made between EBV infections, chromosomal breakpoints, and cancerous mutations rely heavily on correlative data without sufficient causal evidence. A more cautious interpretation of the results, highlighting the need for further experimental validation, would strengthen the manuscript.

#### Consideration of Alternate Hypotheses

The paper could benefit from a more balanced discussion of alternative hypotheses explaining the observed data. For instance, the role of other environmental, genetic, or lifestyle factors in breast cancer development is not adequately considered. Acknowledging and discussing these potential confounders would provide a more comprehensive understanding of the complex etiology of breast cancer.

#### References and Current Literature

While the paper cites a significant amount of relevant literature, it sometimes overlooks recent studies that could either support or challenge the proposed hypotheses. Incorporating a more current and diverse range of references would enhance the paper’s relevance and credibility.

### Recommendation for Discussion Inclusion

To broaden the discussion and contextualize the findings within the broader research landscape, it is recommended to include the following article in the discussion section.

Al-Awaida W, Al-Ameer HJ, Sharab A, Akasheh RT. Modulation of wheatgrass (*Triticum aestivum Linn*) toxicity against breast cancer cell lines by simulated microgravity. Curr Res Toxicol. Sep 19, 2023;5:100127. [doi: 10.1016/j.crtox.2023.100127] [Medline: 37767028]

Incorporating this article could provide valuable insights into innovative approaches for studying cancer therapies. Specifically, the effects of simulated microgravity on the efficacy of natural compounds like wheatgrass against breast cancer could open up new avenues for research on the environmental and physical conditions affecting cancer treatment outcomes. Discussing this study would enrich the manuscript by introducing the concept of microgravity as a novel factor influencing cancer cell behavior and therapy resistance, thereby offering a broader perspective on cancer research methodologies and therapeutic strategies.

## Round 2 Review

### General Comments

This paper tests the idea that EBV infections can help cause breast cancer by weakening the body’s defenses against cancer. The study uses bioinformatics to compare chromosome breakpoints in breast cancer to those in cancers known to be caused by EBV. The results show that EBV might play a role in breast cancer by damaging important cell functions.

### Specific Comments

#### Major Comments

The methods section needs more details about how the datasets were chosen and combined.

The discussion should explain more about how EBV might cause the chromosome breaks and rearrangements seen in breast cancer.

More data or references are needed to support the idea that EBV helps breast cancer spread to other parts of the body.

#### Minor Comments

Adding more references would strengthen the sections that talk about how EBV affects breast cancer.

Figures and tables should be clearly mentioned in the text to help readers follow the data.

Some parts of the manuscript need clearer writing and better organization, especially where complex bioinformatics results are explained.

The abstract should be revised to clearly highlight the main findings and why they are important.

Make sure all abbreviations are defined when they are first used to help readers understand the text better.

## Abbreviations

EBV: Epstein-Barr virus
